# Interactions between physical activity, diet, and gut microbiota in gastrointestinal health: celiac disease as a model in children and athletes

**DOI:** 10.3389/fspor.2026.1940762

**Published:** 2026-10-21

**Authors:** Alessia Montano, Alessandro Paolini, Cristina Felli, Stefania Paola Bruno, Antonella Baldassarre, Semih Sandal, Andrea Masotti

**Affiliations:** 1 Emergency and Internal Medicine Department, Isola Tiberina Hospital – Gemelli Isola, Rome, Italy; 2 Research Laboratories, Bambino Gesù Children's Hospital IRCCS, Rome, Italy; 3 Department of Pediatric Gastroenterology, Ankara Training and Research Hospital, Ankara, Türkiye

**Keywords:** athletes, celiac disease, children, gastrointestinal health, gluten-free diet, gut microbiota, mediterranean diet, physical activity

## Abstract

Physical activity, diet, and gut microbiota are key determinants of gastrointestinal physiology, immune regulation, and metabolic homeostasis, collectively influencing gastrointestinal health, physical performance, and long-term wellbeing. Although regular physical activity generally promotes intestinal function and systemic health, excessive exercise, nutritional imbalance, and underlying gastrointestinal (GI) disorders may adversely affect gastrointestinal integrity and athletic performance. Within this context, celiac disease (CeD) represents a unique clinical model for understanding the complex interactions among physical activity, dietary interventions, gut microbiota, and gastrointestinal health in both children and athletes. This clinical review provides a comprehensive overview of current evidence regarding these interactions, focusing on gastrointestinal physiology, exercise-related GI disorders, bone health, dietary strategies, and gut microbiota modulation. Current evidence indicates that appropriately prescribed physical activity improves gastrointestinal function, immune regulation, body composition, skeletal health, and quality of life, partly through favorable modulation of the gut microbiota, whereas prolonged or poorly individualized high-intensity exercise may impair gastrointestinal integrity through splanchnic hypoperfusion, increased intestinal permeability, oxidative stress, and transient dysbiosis. A Mediterranean-style gluten-free diet appears to improve nutritional adequacy and musculoskeletal health, while a short-term low-fermentable oligosaccharides, disaccharides, monosaccharides, and polyols (low-FODMAP) diet may relieve persistent irritable bowel syndrome-like symptoms in selected individuals despite strict gluten-free diet adherence. In children with celiac disease, integrating individualized physical activity with optimized dietary management supports growth, skeletal development, and long-term health while addressing the specific physiological demands of physically active children and young athletes. Overall, celiac disease provides a clinically relevant model for understanding how physical activity, diet, and gut microbiota interact to influence gastrointestinal health, highlighting the importance of individualized lifestyle strategies that integrate appropriate exercise with nutritional optimization to improve gastrointestinal function, musculoskeletal health, and quality of life.

## 1 Introduction

Physical activity is widely recognized as a fundamental component of a healthy lifestyle and plays a pivotal role in the prevention and management of several chronic diseases. Beyond its well-established systemic benefits, physical exercise exerts direct and indirect effects on the gastrointestinal (GI) tract by influencing motility, intestinal permeability, immune regulation, and gut microbiota composition (1, 2). While regular low-to moderate-intensity physical activity is generally associated with improved GI function and reduced inflammation, intense or prolonged exercise may induce GI symptoms, particularly in susceptible individuals or in the presence of underlying GI disorders (3).

GI disorders are common among physically active individuals and athletes and represent a frequent cause of discomfort, impaired performance, and reduced quality of life. These symptoms arise from a complex interplay of physiological, mechanical, nutritional, and neuroendocrine factors, including alterations in splanchnic blood flow, autonomic nervous system balance, and dietary practices surrounding exercise (2). Consequently, understanding the bidirectional relationship between physical activity and GI health has become increasingly relevant in both clinical and sports medicine settings.

Building upon this physiological framework, celiac disease (CeD) emerges as an exceptionally fitting clinical model for investigating exercise–gut interactions. Because the core pathophysiology of CeD—namely, immune-mediated mucosal injury and compromised intestinal barrier function—directly mirrors the vulnerabilities exposed during strenuous exercise, it provides a powerful lens through which to study these dynamics. As a chronic immune-mediated enteropathy triggered by dietary gluten in genetically predisposed individuals, CeD is characterized by intestinal mucosal damage, malabsorption, and a wide spectrum of extraintestinal manifestations (4). Despite strict adherence to a gluten-free diet, a substantial proportion of patients continue to experience GI symptoms, impaired quality of life, nutritional deficiencies, and compromised bone health (4). These challenges may be further amplified in physically active individuals and athletes, who face increased metabolic demands and specific nutritional constraints.

Emerging evidence suggests that physical activity may exert beneficial effects in individuals with celiac disease when appropriately prescribed, contributing to improvements in body composition, skeletal health, inflammatory status, and psychological wellbeing (3). At the same time, inappropriate exercise intensity or insufficient nutritional support may exacerbate GI symptoms or hinder optimal disease management. In parallel, growing interest has focused on the role of gut microbiota modulation through exercise and diet, highlighting potential mechanisms linking physical activity, intestinal homeostasis, and systemic health (1).

While previous reviews have separately examined the effects of physical activity on GI disorders, the role of exercise in gut microbiota modulation, or clinical aspects of CeD, no comprehensive review has integrated these interconnected dimensions within a single conceptual framework using CeD as a clinical model. In particular, important uncertainties remain regarding how physical activity, dietary interventions (including gluten-free and Mediterranean-style diets), and gut microbiota interact to influence GI health, symptom burden, bone health, and quality of life in individuals with CeD, especially in physically active children and athletes. Furthermore, available evidence is fragmented, and the benefits and potential risks of exercise in CeD, including the influence of exercise intensity and training load, remain incompletely characterized. Therefore, the purpose of this review is to synthesize evidence across these traditionally separate fields (Table 1), thereby providing a novel and clinically relevant perspective on the role of integrated lifestyle strategies in the management of CeD.

**Table 1:** Key Scientific Studies.

Study/Reference	Design	Study Population	Intervention/Exposure	Key Outcomes/Findings
2. PHYSICAL ACTIVITY AND GASTROINTESTINAL PHYSIOLOGY				
Dose-Response of Exercise (1, 3, 5)	Review & Expert Consensus	Competitive athletes, amateur exercisers, and patients with GI conditions.	Varied training volumes, intensities, and duration of physical exercise.	Moderate exercise is protective and improves gastrointestinal symptoms. In contrast, high-intensity or prolonged training suppresses the immune system and impairs gut function.
Postural & Mechanical Stress (6, 7, 8, 12)	Prevalence & Observational Reviews	Endurance athletes (runners, cyclists, swimmers, triathletes).	Postural stressors (vertical running impacts vs. static forward-flexed cycling position) and mechanical jarring.	Cyclists experience more upper gut symptoms like nausea and reflux due to forward-flexed posture. Runners experience more lower gut symptoms like diarrhea and cramps from mechanical stress and reduced gut blood flow.
Pathophysiology of GI Distress (2, 11, 13, 15, 16, 17)	Physiological & Mechanistic Studies	Athletes undergoing strenuous exercise, and experimental animal models.	Acute high-intensity exercise vs. low-intensity activity.	Intense exercise diverts blood flow from the gut, causing temporary oxygen depletion and an increased risk of leaky gut. Moderate exercise improves stomach emptying and releases beneficial proteins that protect the gut lining.
Nutritional & Carbohydrate Guidelines (18, 19, 20, 21)	Position Stands & Nutritional Interventions	Endurance and ultra-endurance athletes.	Pre-race fiber/fructose reduction; intra-exercise carbohydrate intake of 30–60 g/h (or up to 90g/h using 2:1 maltodextrin-to-fructose ratio) and energy gels (50g carbohydrates every 50 mins).	A 2:1 ratio of maltodextrin and fructose is optimal to minimize digestive stress. Consuming more than 60 grams of carbohydrates per hour of a single sugar causes gut distress and fluid accumulation.
3. PHYSICAL ACTIVITY IN GASTROINTESTINAL AND INFLAMMATORY CONDITIONS				
Anti-inflammatory Mechanisms (22, 23, 25, 26, 27, 31, 32)	Immunological & Clinical Reviews	Patients with chronic inflammatory GI disorders (IBS, IBD, diverticulitis).	Regular, moderate physical training vs. sedentary lifestyle.	Regular exercise releases muscle-derived proteins that fight systemic inflammation. It also increases the diversity of beneficial gut bacteria that produce anti-inflammatory molecules like butyrate.
Physical Activity for IBS (42)	Cochrane Systematic Review	Adults with Irritable Bowel Syndrome (IBS).	Various structured physical activity programs.	Regular physical activity significantly reduces the severity of symptoms in irritable bowel syndrome and improves overall quality of life.
Walking & Aerobic Exercise in IBS (43, 53, 54)	Structured Intervention Trials (e.g., StepFit-18K)	IBS patients in Southern Italy.	3-month (12-week) moderate aerobic exercise or structured walking program.	A three-month walking or aerobic program improves physical fitness, relieves bloating and abdominal pain, and reduces psychological stress.
Pediatric Mind-Body Interventions (56, 57, 58)	Randomized Controlled & Pilot Trials	Children and adolescents with IBS or functional abdominal pain (FAP).	Structured yoga programs (integrating physical movement with deep breathing and stress reduction).	Structured yoga reduces the frequency and severity of abdominal pain in children. It also significantly improves quality of life by combining physical movement with stress reduction.
Exercise Safety in adult IBD (29)	Randomized Controlled Cross-over Trial	Patients with quiescent Inflammatory Bowel Disease (IBD).	Moderate-intensity aerobic and resistance exercise.	Moderate exercise is safe for adults with stable inflammatory bowel disease. It improves physical fitness and muscle mass without causing symptom flare-ups.
Fatigue & QoL in adult IBD (64, 66, 67)	Prospective Controlled Trials	Patients with quiescent or mildly active IBD.	10-week structured physical exercise program or personalized training.	Personalized exercise programs significantly reduce fatigue and increase muscle strength in adults with inflammatory bowel disease, leading to better quality of life.
Pediatric IBD Resistance Training (78, 79, 80)	Clinical & Observational Studies	Children and adolescents with IBD in clinical remission.	Structured, home-based resistance training and moderate-to-vigorous physical activity programs.	Resistance training improves bone density and lean muscle mass in children with inflammatory bowel disease. It also helps reduce bowel symptoms and fatigue.
GERD & Exercise Interactions (85, 91, 92)	Case Reports, Observational Surveys, & Systematic Reviews	Athletic children, endurance runners, rowers, and weightlifters.	Strenuous high-impact exercise, running, jumping, and bending over vs. moderate-intensity recreational activities (walking, swimming, yoga).	Vigorous or high-impact exercise can trigger acid reflux by increasing abdominal pressure. Conversely, moderate exercise like walking or swimming improves digestion and reduces reflux risk.
Diverticular Disease Prevention (94, 95)	Large-Scale Prospective Cohort Studies	Adult men.	High levels of vigorous physical activity (e.g., running) vs. sedentary lifestyle.	Vigorous exercise like running reduces the risk of diverticulitis by 44% and lowers the risk of bleeding. It works by improving bowel movement and reducing intestinal pressure.
4. CELIAC DISEASE (CeD) AS A CLINICAL MODEL				
Celiac Symptom Burden in Athletes (123)	Cross-Sectional Survey	Athletes presenting with symptoms similar to Celiac Disease.	Assessment using the Celiac Symptom Index (CSI) and Health-Related Quality of Life (HR-QoL) scales.	Athletes with untreated celiac symptoms report lower quality of life. The symptom burden negatively affects training, mental toughness, and performance.
GFD Constraints in Ultra-Endurance (127)	Single-Athlete Case Study	A 31-year-old female cyclist with Celiac Disease.	Pre-race nutrition planning and GFD adherence during a K4 (384 km) ultra-endurance event.	Ultra-endurance athletes with celiac disease face high risks of under-fueling during races. Commercial gluten-free foods are often dry, crumbly, and difficult to consume while competing.
Bone Mass in Celiac Women (131)	Longitudinal Observational Cohort Study	Adult women diagnosed with Celiac Disease (CeD).	2 years of monitoring on a strict Gluten-Free Diet (GFD) and evaluation of physical activity.	A gluten-free diet improves bone mineral density in celiac women over two years. However, diet alone is insufficient to fully recover bone health without active exercise.
Resistance Training in Celiac Women (132, 133)	Randomized Controlled Trial	Adult celiac women.	12 weeks of strength training combined with a personalized Gluten-Free Diet (GFD + E) vs. GFD alone.	Combining a gluten-free diet with strength training significantly reduces body fat and increases muscle mass in celiac women. It also improves muscle strength and mental well-being.
Sex-Gender Differences in CeD (134)	Longitudinal Adult Cohort Study	Adult celiac patients at diagnosis and during GFD follow-up.	12–30 months of adherence to a strict GFD.	At diagnosis, celiac women present more often with anemia and gut symptoms, whereas men present more often with osteoporosis. Exercise programs should be tailored to individual risks rather than gender.
Anti-inflammatory Synergy (135)	Clinical Intervention Study	Patients with Celiac Disease.	Aerobic exercise combined with dietary fish oil supplementation.	Combining aerobic exercise with fish oil supplements significantly decreases key inflammatory markers in celiac disease patients, showing a synergistic benefit.
Pediatric Celiac Bone Status (137)	Systematic Review & Meta-Analysis of 12 Studies	Children and adolescents diagnosed with Celiac Disease vs. healthy peers.	Meta-analysis comparing skeletal and growth parameters.	Children and adolescents with celiac disease have lower bone mineral density, less bone content, and shorter stature than healthy peers.
Synergy of Lifestyle in Pediatric CeD (138, 183)	Observational Cross-Sectional Study	Children with Celiac Disease following a GFD vs. healthy controls.	Adherence to the Mediterranean Diet (MD) and Physical Activity (PA) levels.	Adhering to the Mediterranean diet and engaging in vigorous exercise significantly improves bone quality and muscle mass in celiac children.
5. MODULATORS OF EXERCISE RESPONSE IN CELIAC DISEASE				
Microbiome of Professional Athletes (148, 156)	Cross-Sectional Comparative Study	Professional rugby players vs. sedentary controls (lean and BMI-matched).	Elite high-volume athletic training and high-protein, high-calorie athletic diets.	Professional athletes show higher gut microbial diversity and more pathways for producing beneficial short-chain fatty acids. However, high protein intake is a major confounding factor.
Exercise in Sedentary Obese Cohorts (158)	6-Month Randomized Controlled Trial	Overweight or obese sedentary subjects.	Structured exercise training (vigorous-intensity group vs. sedentary control).	Vigorous exercise training significantly increases gut microbial diversity in sedentary, obese adults within three months.
Reversibility of Microbiota Changes (32)	6-Week Exercise Training and 6-Week Washout (Sedentary)	Lean and obese previously sedentary humans.	Supervised progressive endurance exercise (3 sessions/week, progressing from 30 to 60 mins and 60% to 75% heart-rate reserve).	Exercise improves the gut microbiota and increases anti-inflammatory butyrate levels. However, these benefits disappear and return to baseline if physical activity is stopped.
Active Lifestyle & Microbial Diversity (159)	Cross-Sectional Observational Study	Active women (meeting standard guidelines) vs. sedentary women.	Spontaneous physical activity levels and habitual diets.	Active women show a significantly higher abundance of beneficial, health-promoting gut bacteria than sedentary women.
Microbiome of Elite Soccer Players (169)	Cross-Sectional Characterization Study	Elite professional soccer players vs. sedentary controls.	Standard professional training regimens vs. sedentary lifestyle.	Elite soccer players have an enriched gut microbiome with higher amounts of beneficial bacteria like Phascolarctobacterium and Prevotella.
Endurance Metagenome Adaptations (170)	6-Week Endurance Exercise Intervention	Overweight, sedentary women.	Endurance training while maintaining a habitual diet.	Endurance training increases the beneficial gut bacteria Akkermansia and decreases inflammatory Proteobacteria, even without changing dietary habits.
Competitive Cyclists Metagenome (171)	Microbiome Community Profiling	Competitive cyclists.	High-intensity endurance training and metabolic lifestyle.	Competitive cyclists show a high abundance of the gut bacteria Prevotella, which increases with higher training volume and improves amino acid metabolism.
Performance-Enhancing Microbes (174)	Meta-omics Analysis & Mouse Models	Elite marathon runners and experimental mouse models.	Post-marathon microbiome isolation and inoculation into mice.	The gut bacterium Veillonella atypica blooms after strenuous exercise and converts lactic acid into propionate, which directly boosts athletic endurance.
Nutritional Adequacy of GFD (182, 183)	Nutritional Surveys & Food Frequency Questionnaires (FFQ)	Children and adolescents with Celiac Disease on a GFD.	Assessment of long-term GFD (>6 months) vs. short-term GFD (<6 months) and healthy controls.	Children on a gluten-free diet often lack folate, iron, magnesium, and selenium. Standard commercial gluten-free products are frequently high in sugar and deficient in essential micronutrients.
Efficacy of Low-FODMAP Diet (185, 186)	Systematic Review & Randomized Controlled Trial	Treated celiac patients suffering from persistent IBS-like symptoms despite GFD adherence.	Short-term moderately low-FODMAP diet (LFD).	A short-term low-FODMAP diet significantly reduces persistent gut pain, bloating, and gas in treated celiac patients. The symptoms are caused by carbohydrate malabsorption rather than gluten.
Microbiome Impact of LFD (188, 189)	Randomized Controlled Trial	Treated celiac patients with persistent GI symptoms.	Low-FODMAP diet restriction.	A low-FODMAP diet reduces beneficial gut Bifidobacteria and alters fatty acid production. This makes professional supervision and a structured food reintroduction phase essential.

A structured mapping of research evidence on physical activity, dietary interventions, gut microbiota, and gastrointestinal health in Celiac Disease (CeD), pediatric populations, and athletes.

## 2 Physical activity and GI physiology

### 2.1 General overview and the dose-response relationship

Patients with GI diseases can experience some difficulties to perform physical activity, although implementing the appropriate level and combination of exercise is generally beneficial (1). GI symptoms typically originate from three main causes: physiological, mechanical, or nutritional factors. Regular and moderate exercise has been demonstrated to have a well-established protective effect against GI disorders, that generally lead to an improvement of symptoms. Conversely, high-intensity or prolonged endurance training can significantly impair GI function. Moreover, heavy training regimens and extended periods of intense exercise are linked to the suppression of immune system functions (5). These exercise-related GI responses reflect a dose-dependent continuum, in which moderate physical activity is generally protective, whereas excessive intensity or duration may predispose susceptible individuals to functional impairment.

### 2.2 Postural and sport-specific mechanical stressors

The GI tract is conventionally divided into four major regions: the upper GI tract (from the esophagus to the stomach), the hepatobiliary system, the small intestines, and the colon. The physiological responses to exercise vary across these regions and depend on the type of sport that is practiced. In fact, the posture adopted by the athlete during the physical activity influences its final effect. This divergence likely reflects sport-specific mechanical stressors and posture-related alterations in intra-abdominal pressure (6).

### 2.3 Physiological mechanisms of exercise-induced GI distress in runners and cyclists

The high prevalence of GI symptoms among endurance athletes—ranging from 30% to over 90% in extreme events—has long been recognized in sports medicine (1, 2). Although both runners and cyclists frequently report digestive distress, the nature, severity, and exact triggers of these symptoms vary significantly between the two disciplines. Understanding these differences requires examining the underlying physiological, biomechanical, and postural mechanisms.

When comparing cycling and running, upper GI symptoms such as nausea, vomiting, and gastroesophageal reflux are more prevalent in cyclists. In contrast, runners more frequently experience lower GI symptoms, including diarrhea, cramping, bleeding, and flatulence (6, 7). Numerous potential pathophysiological factors affect lower GI tract function in endurance athletes, which may contribute to or complicate the condition commonly known as “Runner's Diarrhea” (8). Due to the static position of the athlete, swimming and cycling appear to be associated with a lower frequency of GI disorders compared to running. Runners are particularly susceptible to these negative effects. The severity of GI symptoms is directly proportional to the intensity of the exercise. These symptoms, which can affect the upper abdomen, lower abdomen, or lateral flanks, often necessitate small pauses, complete withdrawal from the race, or at least a temporary reduction in intensity (9). However, runners can mitigate their risks through appropriate fluid intake and by limiting the use of non-steroidal anti-inflammatory drugs (NSAIDs) (10).

GI symptoms which occur during both high-intensity running and cycling are mainly due to splanchnic hypoperfusion. When exercising strenuously, blood flow is diverted from the GI tract to the working muscles and to the skin, leading to temporary intestinal ischemia. This may damage the intestinal barrier and affect the absorption of nutrients and fluid, thereby causing increased permeability, diarrhea, cramping, and malabsorption (9, 11). Running also subjects the GI tract to a considerable amount of mechanical stress as a result of the repeated vertical oscillations and impacts. This kind of jostling can speed up the movement through the colon, increase the shear stress on the mucous membrane, and lead to lower GI symptoms such as abdominal cramps and intestinal bleeding (7).

In cyclists, GI symptoms are more greatly affected by posture and intra-abdominal pressure; the prolonged forward-flexed position can compress the abdomen, lead to an increase in reflux, and hinder esophageal clearance, thus contributing to GERD and other upper GI symptoms (7, 12).

Finally, endurance exercise causes hormonal and biochemical changes which influence GI motility and secretion, and intense exercise delays the emptying of the stomach. Together with the mechanical effect, nausea, retching and vomiting are therefore especially frequent among runners (7, 13).

### 2.4 Physiological and pathophysiological mechanisms

The physiological responses during physical exercise are governed by the balance between the sympathetic and parasympathetic nervous systems. This autonomic shift represents a central mechanism linking exercise intensity to GI symptom generation. During typical exercise, parasympathetic activity decreases while sympathetic activity increases, leading to vasoconstriction. The reduction in parasympathetic activity results in decreased esophageal peristalsis and lower esophageal sphincter tone, which can cause gastroesophageal reflux. This downregulation of neuroendocrine function, blood flow, and effective nutrient absorption may also be a contributing factor to overtraining. GI problems are a common occurrence, particularly among endurance athletes, and represent the most frequent cause of underperformance in competitive events. A primary contributor to the development of GI symptoms is the significant reduction in mesenteric blood flow during intense exercise, which is considered a key pathophysiological driver of exercise-induced GI dysfunction. These symptoms affect both competitive and amateur athletes. They are more frequent in athletes who rapidly increase their training volume, in those with a history of such issues, and in individuals suffering from colitis or gastritis. Furthermore, symptoms occur more often during competitions than in training, especially when the intensity is high (13).

Regardless of the specific sport, physical exercise influences intestinal health through several key mechanisms, including motility and gastric emptying, intestinal permeability, and the neuroendocrine and immune axes. Specifically, acute exercise affects gastric motility in a dose-response manner related to intensity (2). Low-intensity exercise generally accelerates the rate of gastric emptying, whereas high-intensity exercise can delay it and inhibit acid production (2). High-intensity exercise, particularly when prolonged and combined with heat stress, can induce splanchnic hypoperfusion (intestinal ischemia), potentially causing transient intestinal injury and increased permeability (14, 15). These effects, however, are typically temporary and benign (3). Overall, these findings support the concept that GI responses to exercise follow a clear intensity-dependent pattern. Finally, physical exercise modulates the balance of the autonomic nervous system, regulating the sympathetic and parasympathetic functions that innervate the GI tract and promotes the release of myokines (such as Irisin and IL-6), which support immune system modulation and the maintenance of the intestinal epithelial barrier (2, 3, 16, 17).

### 2.5 Nutritional strategies and carbohydrate management

To prevent or reduce GI disorders, several key recommendations exist for the pre-race period and competition day. From a practical perspective, in the two days leading up to the race, it is advisable to minimize the intake of vegetables and fruit. Their high fiber and fructose content can increase water retention in the intestine, thereby increasing bowel activity. Reducing or eliminating coffee is also important, as caffeine can stimulate intestinal activity, especially when pre-competition tension is already high. Fatty dairy products, whole yogurt, cappuccino, and ice cream should be avoided the day before and on race day, as they require a long time to digest, slow gastric emptying, and increase intestinal activity. Regarding carbohydrate intake, sugar intake also plays a crucial role, and some of them can exacerbate GI disorders. An excess of simple sugars can increase intestinal activity, which is already stimulated by the mechanical vibrations of running. Recent scientific studies suggest that mixtures based on maltodextrin and fructose in a ratio not exceeding 2:1 appear optimal for reducing the risk of GI stress while maximizing absorption efficacy. Generally, it is recommended to choose carbohydrates that are rapidly oxidized to prevent their accumulation in the intestine. Most carbohydrate drinks for endurance athletes use glucose and sucrose as primary energy sources, while others use maltodextrins or starches that are rapidly utilized by muscle. All these carbohydrates are equally beneficial in terms of carbohydrate delivery. Guidelines published by the American College of Sports Medicine (ACSM) from 2007 to 2009 demonstrated that during an exercise that lasts more than one hour, athletes should consume 30–60 grams of carbohydrates at hourly rates as the maximum oxidation rate is 1 g/min (60 g/h), regardless of the carbohydrate type (18). Therefore, consuming more than 60 g/h, even of glucose (e.g., 100 g/h), is not only inefficient for energy gain but can also be detrimental by causing intestinal stress. The use of specific products and beverages in the form of “energetic gels” has appeared in recent years and they are widely consumed. The practical solution is to consume one gel (typically containing about 50 g of carbohydrates) every 50 minutes of exercise (19, 20).

### 2.6 Clinical management and lifestyle interventions

To optimize clinical management, a thorough inventory of the patient's dietary, herbal, and supplement intake, including sports drinks, gels, caffeine, alcohol, tobacco, and dietary fiber, is essential. A food diary is often helpful, as patients may struggle to recall specific dietary details. Consultation with a dietitian or nutritionist may be necessary (8). Appropriate nutritional choices can minimize the risk of GI discomfort during exercise by ensuring rapid gastric emptying, efficient absorption of water and nutrients, and adequate perfusion of the splanchnic vasculature (13). Lifestyle modifications are the first step to improving upper GI disorders in athletes. Regarding nutritional adjustments, avoiding solid food and carbohydrate drinks before and during exercise can reduce GI complications by optimizing absorption and facilitating gastric emptying (21). These general physiological mechanisms provide the foundation for understanding how physical activity may differentially influence specific gastrointestinal and inflammatory diseases.

## 3 Physical activity in gastrointestinal and inflammatory conditions

### 3.1 General effects of physical activity on gastrointestinal and inflammatory diseases

In recent years, the significance of exercise as a first-line, non-pharmacological intervention for several chronic diseases has become increasingly clear. Physical activity is now widely recognized as a key modifiable factor influencing GI health across a broad spectrum of diseases.

Many GI disorders/diseases, such as Irritable Bowel Syndrome (IBS), Inflammatory Bowel Disease (IBD)—including its two main forms, ulcerative colitis (UC) and Crohn's disease (CD)—and diverticulitis, are characterized by chronic inflammation. This is characterized by the infiltration of inflammatory cells and high concentrations of inflammatory mediators in the systemic circulation, potentially causing pathological disorders in distant organs (22). Well-documented observations show that physical activity is inversely correlated with systemic low-level inflammation, suggesting that the anti-inflammatory effects induced by regular exercise are responsible for many beneficial health outcomes in patients with chronic conditions (23).

The anti-inflammatory effects of exercise are mediated through multiple interconnected mechanisms involving immune regulation, skeletal muscle signaling, and gut microbiota modulation (5). Contracting skeletal muscles release myokines, such as interleukin-6 (IL-6) and irisin, which stimulate anti-inflammatory pathways and contribute to the regulation of both innate and adaptive immune responses (24, 25). Unlike chronic elevations of IL-6 observed in inflammatory diseases, the transient increase induced by exercise promotes the release of anti-inflammatory mediators, including interleukin-10 and interleukin-1 receptor antagonist, while suppressing the production of pro-inflammatory cytokines such as tumor necrosis factor-α (26). These adaptations contribute to the reduction of chronic low-grade inflammation frequently observed in gastrointestinal disorders (27).

Regular physical activity also influences immune cell activity by improving immunosurveillance and promoting a more balanced immune profile (27). Exercise has been associated with enhanced regulatory immune responses, improved lymphocyte function, and reduced infiltration of pro-inflammatory immune cells into peripheral tissues (28). These effects may be particularly relevant in chronic inflammatory gastrointestinal diseases, where dysregulated immune responses play a central pathogenic role (29).

Furthermore, increasing evidence suggests that part of the anti-inflammatory effect of exercise is mediated through the gut microbiota (30, 31). Regular physical activity promotes greater microbial diversity and increases the abundance of short-chain fatty acid (SCFA)-producing bacteria, including Faecalibacterium, Roseburia, Lactobacillus, and Bifidobacterium species (31, 32). SCFAs, particularly butyrate, support intestinal epithelial integrity, reinforce barrier function, and exert local and systemic immunomodulatory effects (33). Through these mechanisms, exercise-induced microbiota adaptations may contribute to the maintenance of intestinal homeostasis and attenuation of chronic inflammation in gastrointestinal diseases (32, 34).

However, the relationship between exercise and inflammation is nuanced: while the circulating levels of inflammatory molecules are directly proportional to the intensity and duration of exercise, with prolonged, intense activity potentially compromising the immune system for several hours, regular training generally increases resistance to infections (35–37).

Moderate-intensity physical activity consistently emerges as a beneficial intervention in gastric diseases by supporting gut health, modulating the microbiome, and improving psychological wellbeing (15, 17–20, 31, 38–40). However, the strength of evidence varies across conditions. While several systematic reviews and randomized controlled trials support the beneficial effects of exercise in disorders such as IBS and IBD, evidence for other gastrointestinal conditions often derives from observational studies or expert opinion, limiting the certainty of current recommendations. Beyond dietary influences, physical exercise is a cornerstone for the prevention of chronic diseases, making it essential to understand the mechanisms through which it interacts with these conditions. Accordingly, exercise should be viewed as an integral component of comprehensive disease management strategies. The role of physical exercise as an adjunct therapy is equally critical in pediatric gastrointestinal and inflammatory conditions, mirroring the benefits observed in adults while addressing unique developmental challenges. In younger population, exercise interventions must consider growth, maturation, and disease-specific vulnerabilities.

Although these mechanisms are biologically plausible and supported by experimental and clinical studies, their relative contribution likely differs according to disease type, exercise modality, training intensity, and individual host factors. Further mechanistic studies are therefore needed to clarify the pathways through which physical activity influences chronic gastrointestinal inflammation.

### 3.2 Athletes with gastrointestinal and inflammatory conditions

The intersection of high-level physical activity and chronic GI disorders/diseases presents a unique set of challenges and considerations for athletes, whether amateur or elite. While moderate exercise is generally protective of GI health, the intensity and duration typical of competitive sports can exacerbate underlying conditions like Irritable Bowel Syndrome (IBS), Inflammatory Bowel Disease (IBD), Gastroesophageal Reflux Disease (GERD), diverticular disease and other GI disorders/diseases. This dual role of physical activity—as both a therapeutic tool and a potential trigger of symptoms—highlights the need for condition-specific exercise strategies. Importantly, much of the evidence regarding athletes with gastrointestinal disorders is based on observational studies, surveys, case series, and expert recommendations rather than large randomized trials. Therefore, current recommendations should be interpreted with caution and individualized according to the athlete's clinical condition.

### 3.3 Disease-specific considerations in athletes

#### 3.3.1 Irritable bowel syndrome (IBS) in athletes

IBS is a prevalent functional GI disorder defined by symptoms such as abdominal pain, bloating, diarrhea, and constipation (40, 41). Regular physical exercise, particularly aerobic activities like walking, cycling, and swimming, is proposed to alleviate these symptoms (42, 43). Notably, the evidence supporting exercise interventions in IBS is relatively robust, including a Cochrane systematic review and several randomized controlled trials demonstrating improvements in symptom severity and quality of life, although optimal exercise modalities and training volumes remain insufficiently defined (42). Exercise-related symptom improvement is mediated through both physiological and psychosocial mechanisms. Aerobic exercise modulates the gut microbiota (40, 44), notably enhancing the diversity and abundance of beneficial bacteria such as Lactobacillus and Bifidobacterium (45, 46). Furthermore, exercise positively influences the gut–brain axis by reducing stress, anxiety, and depression (38, 47–49), which are common comorbidities associated with IBS (50). Mind-body exercises like yoga and Tai Chi are also effective in symptom alleviation by combining physical activity with stress reduction techniques (43, 47, 51, 52). These interventions are particularly valuable in patients with prominent stress-related symptom patterns. Crucially, while low-to-moderate intensity exercise is highly beneficial (43, 53, 54), exercise programs must be personalized (40), as inappropriate or excessive exercise can sometimes exacerbate IBS symptoms (55). While high-intensity aerobic exercise is generally recommended for adults with IBS, in the pediatric population, mind-body interventions often show the most promising results. Randomized trials have shown that yoga, which combines physical activity with stress reduction techniques, significantly reduces the severity and frequency of abdominal pain and improves QoL in adolescents with IBS and functional abdominal pain (FAP) (56–58). These findings underscore the importance of age-specific exercise strategies.

IBS symptoms are highly prevalent among athletes, often manifesting as exercise-induced GI symptoms (Ex-GIS) such as bloating, cramping, and diarrhea, particularly in endurance sports (59). These symptoms can significantly impair training quality and competitive performance. The relationship is complex: while low-to-moderate intensity aerobic exercise is a recognized treatment for IBS in the general population (42), the high-intensity nature of competitive training can trigger or worsen symptoms. This paradox underscores the importance of carefully balancing exercise intensity and volume. Athletes with IBS often benefit from specific nutritional strategies, such as the low-FODMAP diet, tailored to their training load, to manage symptoms without compromising caloric intake (60). Such individualized dietary approaches are particularly relevant in endurance athletes with high energy demands.

#### 3.3.2 Inflammatory bowel disease (IBD) in athletes

For patients with IBD, encompassing Crohn's disease (CD) and ulcerative colitis (UC) (61, 62), regular exercise regimens are considered essential (29, 63). Exercise represents a safe and effective adjunct therapy when disease activity is adequately controlled. Low-to-moderate intensity exercise, including both aerobic and resistance training, has been shown to improve the quality of life (29, 64, 65), reduce fatigue (66–68), and help counteract IBD-related complications (29), particularly in patients in remission or with mild active disease (64). Physical exercise reduces systemic inflammation (69, 70). Skeletal muscle contractions release anti-inflammatory myokines that influence the intestinal microenvironment and modulate immune function (69–72). Exercise also modulates the gut microbiota (63, 73), helping to restore diversity and alter the patterns of dysbiosis observed in IBD (32, 63). These mechanisms collectively contribute to improved disease control. Furthermore, physical activity improves cardiorespiratory fitness (67), muscular strength (74), and bone mineral density (75), and important benefit given the risk of bone loss in IBD. Finally, it improves psychological health and stress management abilities (76, 77). For children and adolescents with IBD, regular, moderate-to-vigorous physical activity is essential. IBD patients are at risk of low bone mineral density (BMD) and reduced muscle mass due to chronic inflammation and corticosteroid use. Studies have demonstrated that a structured, home-based resistance training program significantly improves BMD and lean body mass (LBM) in children and adolescents with IBD in clinical remission (78, 79). These findings emphasize the long-term musculoskeletal benefits of early exercise interventions. Furthermore, PA helps mitigate common IBD-related symptoms such as fatigue and psychological distress, leading to improved quality of life (QoL) (80, 81). Most evidence in IBD derives from randomized trials and systematic reviews conducted in patients with quiescent or mildly active disease. Consequently, the safety and efficacy of exercise during periods of moderate-to-severe disease activity remain less well established.

Athletes with IBD (Crohn's disease or Ulcerative Colitis) face the dual challenge of managing their disease while maintaining peak performance. Exercise is generally encouraged for its benefits on QoL, fatigue reduction, and psychological wellbeing (82). In clinically stable patients, regular physical activity is considered a key component of comprehensive disease management. However, intense training must be carefully managed, especially during flares or periods of high disease activity. The primary risk for endurance athletes with IBD is the potential for exercise-induced splanchnic hypoperfusion (reduced blood flow to the gut), which can lead to transient intestinal injury or, in rare cases, ischemic colitis, even in recreational athletes (42). This risk emphasizes the need for close monitoring and individualized exercise prescriptions. Despite these risks, many professional athletes successfully manage their IBD, highlighting the importance of personalized medical and nutritional support (82). Multidisciplinary care plays a central role in enabling safe sports participation.

#### 3.3.3 Gastroesophageal reflux disease (GERD) in athletes

GERD is a common GI motility dysfunction characterized by the irregular reflux of stomach contents into the esophagus, causing symptoms such as heartburn, regurgitation, chest pain, and difficulty swallowing (3, 83, 84). Exercise-related reflux is a frequent concern in both adult and pediatric populations. The relationship between physical activity (PA) and GERD is complex, as exercise can both trigger and alleviate symptoms depending on the activity's type, intensity, and timing (3, 85). High-intensity physical activity and prolonged strenuous exercise can exacerbate reflux episodes (3, 85). This worsening is linked to a decrease in blood flow (splanchnic hypoperfusion) to the GI tract and the activation of adrenergic receptors (86). Moreover, vigorous exercise, especially high-impact activities (like running or jumping) or activities involving bending over or lying flat, can worsen GERD symptoms (85, 87). These activities increase intra-abdominal pressure, which can impair the function of the Lower Esophageal Sphincter (LES) or force gastric contents upward into the esophagus, thereby aggravating reflux (85, 88). Conversely, higher levels of recreational physical activity are associated with a reduced risk of developing GERD and esophageal adenocarcinoma (3, 89). Intermediate physical activity levels significantly decrease GERD risk among obese individuals (87). Moderate exercise aids in weight management, which is critical since obesity, particularly abdominal obesity, increases intra-abdominal pressure and exacerbates reflux (85, 89). Moderate-intensity physical activity (e.g., walking, swimming, cycling) improves overall digestive health and gut motility, reducing associated symptoms like bloating and indigestion (85). Low-impact, mind-body exercises like yoga and Tai Chi can alleviate stress, a common GERD trigger, by helping regulate the autonomic nervous system (85, 90). In GERD condition, the relationship with exercise remains complex and highly individualized, even in children. While moderate PA is generally encouraged for overall health, strenuous or high-impact activities, particularly those involving bending or increased intra-abdominal pressure, can exacerbate reflux symptoms in susceptible athletic children (91). Therefore, the focus remains on encouraging regular, moderate activity while carefully avoiding specific triggers, emphasizing that exercise prescription must be tailored to the child's specific symptoms and disease activity level.

GERD is significantly more prevalent in athletes, particularly those involved in endurance sports (e.g., running, cycling) and strength sports (e.g., weightlifting), compared to the general population (92). Exercise-related reflux represents a frequent yet often underrecognized cause of impaired athletic performance. The mechanism involves the increase in intra-abdominal pressure during intense exertion, which compromises the function of the Lower Esophageal Sphincter (LES) (6). Studies show that GERD is frequently induced during exercise, especially in runners and rowers (92). Management for the athlete focuses on optimizing the timing and composition of pre-exercise meals, avoiding specific triggers, and selecting lower-impact activities during symptomatic periods. High-level athletes often require pharmacological intervention to maintain training volume (6). Thus, symptom control must be balanced against the demands of high-level training.

#### 3.3.4 Diverticular disease and other GI disorders

Physical exercise is recognized as a crucial non-pharmacological strategy for both the prevention and management of diverticular disease, which includes diverticulosis (the presence of colonic pouches) and diverticulitis (inflammation or infection of these pouches) (3, 85, 93). Several studies indicate an inverse relationship between physical exercise and the risk of GI disorders, including diverticulitis (3, 85). Regular physical activity is associated with a significantly reduced risk of complications related to diverticular disease, such as diverticulitis and diverticular bleeding (85). Vigorous physical activity is particularly protective, leading to a 44% reduction in the risk of diverticulitis [Relative Risk (RR) = 0.66] and a reduction in diverticular bleeding (RR = 0.61) compared to sedentary lifestyles in men (85, 94). These data support the role of physical activity as a preventive public health strategy.

Unlike inflammatory conditions, diverticular disease shows a strong inverse association with physical activity, particularly vigorous exercise. Studies consistently demonstrate that high levels of vigorous physical activity, such as running, significantly reduce the risk of developing diverticulitis and diverticular bleeding in men (94, 95). This protective effect highlights physical activity as a key preventive strategy that allows the maintenance of GI motility and the reduction of intra-colonic pressure. For athletes, this means that maintaining a high level of vigorous activity is a key preventative measure against this condition.

Regular moderate-intensity aerobic exercise enhances intestinal motility (44), improves colonic transit time, and reduces constipation risk (96, 97). Activities like walking and core-strengthening exercises are particularly helpful for improving bowel regularity (96, 97). Regular physical activity is inversely related to the risk of several GI cancers (98), particularly colorectal cancer (99, 100). This protective effect is linked to reduced systemic inflammation (101), improved immune function (102, 103). and positive modulation of the gut microbiota, which promotes SCFA production (100, 104, 105). Moderate-intensity activities like walking, cycling, or swimming can support ulcer management by reducing stress and improving circulation (106, 107), thereby promoting the healing of the GI mucosa (106, 108). High-intensity exercise that increases abdominal pressure should generally be avoided, as it can worsen symptoms (55, 109).

In conclusion, for most GI disorders, the evidence overwhelmingly supports regular, moderate-intensity physical activity as a valuable adjunct therapy due to its holistic benefits on motility, inflammation, microbial balance, and mental health (31, 110). However, the quality of evidence varies substantially across conditions, and further adequately powered randomized controlled trials are needed to define optimal exercise prescriptions, particularly in athletes and pediatric populations. The key determinant of benefit remains the appropriate individualization of exercise intensity, duration, and modality. For high-intensity activity, it consists in implementing personalized strategies to maximize benefits while mitigating risks (i.e., splanchnic hypoperfusion or increased intestinal permeability) (111, 112). Regarding other GI disorders, physical activity is strongly linked to a reduced risk of GI cancers, particularly colorectal cancer. This protective effect is observed even in athletes and is attributed to reduced systemic inflammation, improved immune function, and positive modulation of the gut microbiota (113, 114). These mechanisms further support the systemic benefits of regular physical activity. However, athletes must be mindful that extreme endurance exercise can lead to transient GI issues, such as GI bleeding or ulceration, due to splanchnic hypoperfusion, necessitating careful monitoring and nutritional strategies (2). Accordingly, individualized risk–benefit assessment remains essential.

## 4 Celiac disease as a clinical model

Among GI disorders, celiac disease represents a particularly informative clinical model for studying exercise–gut interactions because it integrates multiple interconnected mechanisms, including intestinal inflammation, altered barrier function, gut microbiota disturbances, nutritional deficiencies, and impaired bone health. Furthermore, the lifelong requirement for a gluten-free diet and the specific challenges faced by physically active individuals and athletes provide a unique framework for understanding how physical activity and dietary interventions jointly influence GI and systemic health.

### 4.1 Athletes with celiac disease

Celiac disease (CeD) is a chronic immuno-mediated enteropathy triggered by dietary gluten—a protein found in wheat, barley, and rye—in genetically susceptible individuals. CeD damages the small intestinal mucosa that lead to villous atrophy, and this in turn reduces absorption of essential nutrients, and subsequent malabsorption (4). From a sports medicine perspective, these pathophysiological alterations acquire particular relevance in athletes, in whom optimal nutrient absorption is critical to sustain training load, recovery, and performance. CeD is often misdiagnosed in athletes and a well-informed sports medicine team can assist in identifying symptoms suggesting CeD, thereby minimizing intestinal damage. As CeD is a chronic autoimmune condition, the only current treatment is a strict adherence to gluten-free diet (GFD) to prevent negative short- and long-term consequences (115). Despite strict GFD adherence, many CeD patients report a poor quality of life (QoL), with up to 30% continuing to experience adverse symptoms, such as GI upset (115). These persistent symptoms may negatively interfere with training continuity, exercise tolerance, and competitive performance. Notably, physical activity rates among women with CeD are frequently very low, with over 50% engaging in only minimal levels of physical activity (PA) (116). This observation is clinically relevant, as reduced physical activity may further aggravate disease-related complications such as impaired bone health, altered body composition, and diminished physical fitness. Research on CeD within the athlete population remains insufficient. Importantly, current evidence in athletes with celiac disease is largely derived from observational studies, surveys, case reports, and expert opinion, while prospective controlled studies remain scarce. Consequently, the true impact of celiac disease on athletic performance, recovery, and long-term training adaptations remains incompletely understood. Even less attention has been paid to study CeD prevalence, symptoms, and potential implications for an athlete's training and performance. Consequently, evidence-based recommendations for the management of physically active individuals with CeD remain limited. One of the most significant challenges posed by CeD is managing the athlete's high metabolic rate and caloric expenditure (117). This challenge is compounded by the fact that the sole treatment is a closely monitored gluten-free diet (GFD), which is critical because micronutrient deficiencies, particularly iron and calcium, are common, and high-caloric carbohydrates are necessary. Thus, athletes with CeD must simultaneously meet elevated energy demands while adhering to strict dietary restrictions. Furthermore, commercially available gluten-free foods often have a high caloric density and can be dry and crumbly, potentially causing stomach distress. This is particularly detrimental for ultra-endurance athletes, as the reduction of these foods can impede the ability to consume sufficient calories. Additionally, the malabsorption caused by the disease can make it difficult to maintain the energy required for strenuous workouts (4, 118). These factors collectively increase the risk of suboptimal fuelling and impaired recovery. Psychological burdens have also been reported, including an increased risk of depression (119), disruptive nutrient alterations that affect the brain, anxiety related to food, and an overall risk of eating disorders that can be very counterproductive to an athlete's training goals (120). Because both compulsory dietary restriction and sport-related pressures may contribute to problematic eating behaviours, screening should be considered when an athlete exhibits restrictive eating beyond the requirements of the GFD, weight or shape concerns, unexplained weight loss, menstrual disturbance, recurrent injury, or declining performance. For adolescent athletes aged 13–19 years, the six-item Disordered Eating Screen for Athletes (DESA-6) provides a brief athlete-specific option validated in both male and female athletes (121). For collegiate and adult athletes, the Eating Disorders Screen for Athletes (EDSA) is another brief instrument developed and validated in male and female athletes from lean and non-lean sports (122). None of these tools have been validated specifically in athletes with CeD. Therefore, a positive screen should be interpreted as an indication of risk rather than a diagnosis and should prompt comprehensive assessment by an appropriately qualified healthcare professional.

The psychological dimension of CeD represents an additional layer of complexity in athletes, where mental resilience is closely linked to performance. The study of Leone and collaborators suggests that athletes who have symptoms similar to CeD have a lower QoL than their peers in the physical, psychological, social, and environmental areas. This is due to a higher Celiac Symptom Index (CSI) score, which suggests that the burden of the disease is multisystem. One explanation could be that the psychological component of the athletic QoL is worse due to the significant impact the CeD symptoms have on the athlete's physical capabilities and mental toughness. Disruptive nutritional alterations that affect social and environmental QoL may be due to symptoms that interfere with daily routines, uncertainty about physical performance, and social withdrawal (123). Together, these findings highlight the broad systemic and psychosocial impact of CeD in physically active individuals. This situation is further explained by the difficulty of achieving sufficient energy and nutrient intake even in healthy individuals; for those with CeD, dietary restrictions introduce major complications. Careful nutritional planning is vital, especially in cases of concomitant anemia, which requires close attention to iron status (124). From a practical monitoring perspective, athletes with CeD should undergo a baseline assessment including a complete blood count, iron status, serum 25-hydroxyvitamin D, albumin, calcium, and phosphate. Abnormal results should be reassessed after correction, generally within 3–6 months, and monitored until normalization; repeat testing at approximately 12 months is appropriate when abnormalities persist or treatment is ongoing. Once clinical stability and biochemical normalization have been achieved, routine follow-up may be extended to every 12–24 months. However, no CeD-specific evidence supports a universal biomarker-monitoring schedule for all athletes, and shorter intervals may be considered according to previous deficiencies, persistent symptoms, dietary adherence, and individual training demands (125, 126).

Such nutritional challenges underscore the need for individualized dietary strategies in athletic populations. A relevant case study involves a 31-year-old female cyclist with CeD who participated in the K4 (384 km) ultra-endurance event. Despite meticulous pre-race nutrition planning, her actual nutrient intake during the event was lower than intended due to the difficulties of adhering to a GFD plan during the race (127). Although illustrative, this report represents a single-athlete experience and therefore cannot be generalized to the broader population of athletes with celiac disease. This case exemplifies the real-world challenges faced by athletes with CeD during prolonged endurance events. Given the increased public awareness and prevalence of celiac disease over the last decade, team physicians must be equipped to both diagnose and manage affected athletes. Sports medicine clinicians need to recognize that CeD can manifest with a wide array of symptoms when evaluating athletes with chronic, often perplexing illnesses. Consequently, CeD must be considered a potential diagnosis when formulating a differential diagnosis (128). Early recognition and multidisciplinary management are essential to optimize both health outcomes and athletic performance.

### 4.2 Physical activity and celiac disease

Exercise is a fundamental component of a balanced lifestyle, and this balance is necessary to maintain the physical wellbeing required for healing. In the context of CeD physical activity represents not only a general health-promoting behavior but also a potential supportive strategy to mitigate disease-related complications. Patients with CeD must take special care to recover, particularly those who may have consumed detrimental foods for a lifetime (129). Specifically, weight training helps achieve a healthy muscle-to-fat ratio and strengthens bones, which may have been weakened by the disease's impact on calcium levels. Osteoporosis and osteopenia (lower than normal bone density) are frequent secondary outcomes of celiac disease (130). Weight-bearing exercises can help prevent further weakening of bones and joints. These aspects are particularly relevant during periods of growth and in physically active individuals. Indeed, one study reported the importance of physical activity in celiac disease and demonstrated that the improvement in bone density following a gluten-free diet was significant after 2 years; however, physical activity levels were frequently low and played a minor role in determining changes in bone mineral density (131). This finding highlights that dietary treatment alone may be insufficient to fully optimize musculoskeletal outcomes. Regular exercise is known to confer numerous physical and psychological health benefits and improve QoL in chronic disease populations (115). Specifically, resistance training, when combined with a personalized GFD, has proven crucial for improving body composition (BC), strength, and GI symptoms in women with CeD (132). The group that followed a personalized GFD and physical exercise (GFD + E) was the only who showed significant body composition improvements, including a decrease in fat mass (FM) and an increase in fat-free mass (FFM) (132). These changes are particularly relevant for athletic performance, metabolic health, and long-term disease management. Furthermore, celiac women in the GFD + E group reported higher scores on the psychological health scale compared to those without intervention (132). They also demonstrated significant improvement in urogenital symptoms and scored higher on the “vigour” subscale of the Profile of Mood States (POMS) questionnaire (132, 133). Together, these findings emphasize the multidimensional benefits of integrating physical activity into the management of CeD.

However, these findings should be interpreted in light of the exclusively female and relatively small study populations. The available resistance-training studies did not directly compare women and men and therefore cannot establish sex-specific differences in exercise responsiveness in CeD (132, 133). Sex-related differences have nevertheless been reported in the clinical presentation of CeD. In a longitudinal adult cohort, women more frequently presented with gastrointestinal symptoms and signs of malabsorption, whereas osteopenia or osteoporosis was less frequent in women than in men at diagnosis (134). After 12–30 months of a GFD, women more frequently had anemia and hypoferritinemia, but gastrointestinal symptom burden no longer differed significantly between the sexes (134). These observations may influence baseline nutritional and skeletal risk assessment, but they do not demonstrate a sex-specific exercise response. Exercise recommendations should therefore not be differentiated solely by sex on the basis of current CeD evidence; instead, individual factors such as nutritional status, bone health, symptoms, menopausal status, and training goals should be considered.

One study also found that aerobic exercise combined with fish oil supplementation promoted a significant reduction in C-reactive protein (CRP) and increased the proportion of individuals with undetectable levels of interleukin-6 (IL-6) in CeD patients (135). These observations support a potential anti-inflammatory role of exercise in CeD.

While these findings are encouraging, they should be interpreted cautiously because most intervention studies conducted in celiac disease involve relatively small sample sizes, predominantly female participants, and short follow-up periods. Consequently, the long-term sustainability and generalizability of these benefits remain uncertain.

### 4.3 Physical activity and bone health in celiac children on a gluten-free diet

Celiac disease is a systemic condition that extends far beyond the GI tract, particularly in children and adolescents. In pediatric populations, this systemic involvement has important implications for growth, musculoskeletal development, and long-term skeletal health. When diagnosed in children, it often happens during child development, a crucial time for bone growth, and this raises some important concerns about bone health. The amount of bone mass that is built up during childhood and adolescence plays a huge role in the overall skeletal health, later in life, especially to decrease the chance of experiencing fractures due to osteoporosis when they reach adulthood (136). Therefore, optimizing bone accrual during these formative years represents a critical preventive strategy. So, it is of fundamental importance paying attention to bone health during these formative years. Fedewa and colleagues discussed this issue in their comprehensive meta-analysis designed to quantify the differences in key skeletal and growth parameters between youth diagnosed with celiac disease and their healthy peers. A careful analysis of twelve different studies showed that young people with celiac disease tend to have poorer bone health. Specifically, they found lower bone mineral content (BMC) and areal bone mineral density (aBMD), as well as shorter stature compared to their peers (137). These findings highlight the early and measurable impact of CeD on skeletal development. While a strict gluten-free diet is the cornerstone therapy for managing celiac disease, it is clear that other modifiable lifestyle factors play a crucial role in optimizing health outcomes, particularly concerning musculoskeletal development. Among these factors, physical activity and dietary quality emerge as key determinants of bone health. Nestares's work examines the impact of adherence to the Mediterranean Diet (MD) and physical activity (PA) on the bone health of children with celiac disease following a gluten-free diet (GFD). The study compared children with a non-celiac control group, finding that the celiac group had lower body weight, lean mass, and bone mineral content. It was found that among children with celiac disease, greater adherence to the MD and more time spent in vigorous PA were associated with better bone Z-scores and greater lean mass, suggesting the importance of carefully monitoring lifestyle. Specifically, lean mass was found to be a factor independently associated with bone mineral density (BMD), underscoring the need for lifestyle interventions to improve bone quality in this population (138). However, many studies assessing physical activity and bone health in pediatric celiac disease are observational in nature, limiting the ability to infer causality. Furthermore, the relative contribution of physical activity, dietary adherence, nutritional status, and pubertal development to skeletal outcomes remains difficult to disentangle. These results support the concept that muscle–bone interactions play a central role in skeletal health among children with CeD. This highlights the crucial need for more in-depth, high-quality research to fully understand the long-term effects of these bone health issues, particularly regarding peak bone mass achievement and potential future risks of osteoporosis and fractures. Longitudinal studies integrating physical activity patterns, dietary adherence, and bone outcomes are essential to inform preventive strategies in this vulnerable population.

Overall, the available evidence suggests that physical activity may represent a valuable adjunctive strategy in the management of celiac disease, with potential benefits extending beyond gastrointestinal symptoms to body composition, bone health, inflammatory status, and quality of life. Nevertheless, the current evidence base remains limited by small study populations, heterogeneous outcome measures, short intervention durations, and a predominance of observational designs. Furthermore, substantial gaps persist regarding the optimal exercise prescription, the impact of physical activity in athletes with celiac disease, and the long-term effects of exercise during childhood and adolescence. Future large-scale randomized controlled trials and longitudinal studies are needed to establish evidence-based recommendations for different celiac disease populations.

Beyond disease-specific outcomes, emerging evidence suggests that the effects of physical activity in celiac disease may be further modulated by gut microbiota composition and dietary patterns.

## 5 Modulators of exercise response in celiac disease

Recent data have suggested that gut microbiota leads to the activation of innate immunity in celiac disease because the interaction between the gut microbiota and the mucosal wall is mediated by the same receptors that can activate innate immunity. For this reason, changes in gut microbiota may activate inflammatory pathways (139, 140). This mechanism provides a biological link between intestinal dysbiosis, immune activation, and disease expression. The integrity and dynamic interaction of gut microbiota are essential to maintaining homeostasis of the human body and a healthy state, while alterations may contribute to the development of diseases, not only those related to the GI tract, but also at metabolic and systemic levels (139, 141). Several studies have shown a close correlation between celiac disease and alterations of the intestinal microbiota during the active phase of the disease (142). However, studies by Di Cagno et al. and Schippa et al. demonstrated that GFD allows only partial recovery of the gut microbiota in celiac disease patients, in both microbiological and metabolomic studies (143, 144). This incomplete restoration suggests that adjunctive lifestyle interventions, including physical activity, may play a role in modulating gut microbial composition. Although studies clearly demonstrate an association between altered intestinal composition and microbiota in CeD patients, a specific “celiac” microbiota signature has not yet been definitively identified, unlike in various inflammatory diseases where the specific role of the intestinal microbiota is recognized (142). Consequently, physical exercise emerges as a potentially modifiable factor capable of influencing gut–immune interactions in CeD.

### 5.1 The role of physical activity in modulating the gut microbiota

A complex bidirectional relationship exists between physical activity and the composition of the gut microbiota (GM) (1, 145). This bidirectional axis is particularly relevant in GI disorders, where microbial composition may influence inflammation, barrier integrity, and symptom burden. The gut microbiota is often associated with a “health-associated” microbial community (146, 147). This modulation can vary significantly based on the intensity and chronicity of the exercise, as well as concurrent dietary patterns (148, 149). Beyond genetics, ethnicity, age, infections, and drugs, nutrition exerts a significant influence on the intestinal microbiota profile (150). However, the most common contributor to microbial changes is human behavior, primarily through diet and exercise (151). Accordingly, interpreting microbiota findings requires consideration of both training characteristics and dietary context. Regular exercise is linked to beneficial changes in the microbiota. Consistent physical activity or a high level of cardiorespiratory fitness (CRF) positively correlates with greater bacterial alpha diversity in the fecal microbiota (148, 152–154). Conversely, reduced microbial diversity has been implicated in various GI disorders (155). Greater microbial diversity is commonly viewed as a marker of ecological resilience and metabolic flexibility. A consistent finding across numerous cross-sectional studies is that highly physically active individuals and athletes exhibit significantly higher gut microbial diversity (alpha diversity/species richness) compared to sedentary controls (146, 148, 149, 152, 156, 157). For example, professional rugby players showed a significantly greater diversity of gut microorganisms, representing 22 distinct phyla, compared to control groups (148). These observations support the concept that high training status is associated with distinct microbial features, although causality cannot be inferred from cross-sectional designs. In intervention studies, exercise training has been demonstrated to induce changes in microbial structure. In a six-month trial involving overweight/obese sedentary subjects, the vigorous intensity group (VIG) experienced an increase in alpha diversity at three months compared to the control group (146, 158). However, other studies focusing on short-term interventions or non-athletes engaging in moderate activity have sometimes reported no changes in alpha diversity (29, 157, 159–161). This variability likely reflects differences in baseline fitness, intervention duration, diet, and analytical methods. These changes may be temporary and require continuous stimulus (32, 145, 146), in fact, when sedentary individuals discontinued a six-week exercise intervention, the microbiota changes largely reverted during a subsequent six-week washout period (32, 145, 146). This reversibility suggests that sustained lifestyle patterns are necessary to maintain exercise-associated microbial adaptations. However, none of the studies have established a minimum maintenance dose of physical activity required to preserve exercise-associated microbiota changes, either in CeD or in the general population. In the study demonstrating reversibility, previously sedentary adults completed supervised endurance exercise three times per week, progressing from 30 to 60 minutes per session and from 60% to 75% of heart-rate reserve; the exercise-associated microbiota changes largely reversed after participants returned to a sedentary lifestyle (32). Because lower frequencies or shorter durations were not compared, this protocol should not be interpreted as a minimum effective dose. In clinical practice, continued adherence to age-appropriate physical activity recommendations may be used as a pragmatic target, while recognizing that these recommendations were developed for general health and have not been validated as a microbiota-specific maintenance threshold in CeD (162). The available evidence therefore supports continuity of physical activity rather than a specific microbiota-directed exercise dose.

An undesirable gut microbiota (GM) composition has been associated with local inflammatory changes that increase gut wall permeability, potentially allowing greater systemic migration of bacterial material; multiple studies link these processes to various chronic diseases (163). The gut microbiota plays a crucial role in inflammatory-based chronic diseases, such as IBS and ulcerative colitis (164, 165). These mechanisms provide a biological rationale for considering exercise-induced microbiota modulation as clinically meaningful. Intestinal homeostasis requires balanced interactions among the gut microbiota, dietary antigens, and the host (166). Environmental factors that disrupt this relationship can contribute to a breakdown in intestinal homeostasis by directly influencing immune and barrier function, as well as the composition of the gut microbiota. This disruption, in turn, can lead to proinflammatory reactions to otherwise innocuous antigens and the development of chronic inflammation (167, 168). Within this framework, physical activity may act as a modulator of host–microbe interactions through immune, neuroendocrine, and metabolic pathways. Physical activity significantly alters the relative abundance of specific bacterial taxa, often favoring species associated with enhanced health and metabolism. In particular, exercise is linked to an increase in beneficial short-chain fatty acid (SCFA)-producing bacteria, such as *Faecalibacterium prausnitzii*, as well as genera like *Lactobacillus* and *Bifidobacterium* (46, 153, 159). Specific genera like *Faecalibacterium prausnitzii* and *Roseburia hominis* are found in higher abundance in active women compared to sedentary women (145, 146, 149, 159). Increased relative abundances of SCFA-producing taxa, including *Clostridiales*, *Roseburia*, *Lachnospiraceae*, and *Erysipelotrichaceae*, were positively correlated with high cardiorespiratory fitness (146, 152). The phylum *Firmicutes* (which includes SCFA-producing families like Ruminococcaceae and Lachnospiraceae) tends to increase with regular physical activity (32, 152). Elite soccer players exhibited a significantly higher prevalence of Genus *Phascolarctobacterium* and species *Phascolarctobacterium succinatutens* and *Prevotella albensis* compared to sedentary individuals (169). The genus *Akkermansia*, which is inversely correlated with metabolic syndrome, is frequently observed as enriched in athletes, such as Irish rugby players (147, 148). Endurance exercise interventions in overweight, sedentary women demonstrated an increase in the relative abundance of *Akkermansia spp.* and a decrease in inflammation-associated *Proteobacteria*. These changes were found to be independent of age, weight, fat percentage, and food intake (145, 157, 170). The abundance of *Prevotella* is associated with endurance exercise programs (148). A high relative abundance of the genus *Prevotella* (including *Prevotella copri*) is often observed in competitive endurance athletes (cyclists and runners) compared to sedentary populations (169, 171, 172). The presence of this genus has been correlated with increased metabolism of branched-chain amino acids (171, 173). The genus *Veillonella* is also detected with elevated abundances in athletes (149). Meta-omics analysis demonstrated that specific performance-enhancing microbes like *Veillonella atypica*, isolated from marathon runners, function via lactate metabolism to produce propionate, which may increase endurance capacity (145, 146, 157, 173, 174). Collectively, these findings suggest that training modality and endurance phenotype may be associated with distinct microbial signatures. The gut microbiomes of professional rugby players demonstrated enrichment for pathways involved in amino acid biosynthesis, carbohydrate metabolism, and SCFA synthesis (148, 156). As a result of exercise, concentrations of short-chain fatty acids (SCFAs), particularly butyrate, increased in the human gut. These levels declined again after the participants reverted to a sedentary lifestyle. Microbiota analysis confirmed that this corresponded to changes in the proportion of microbes that produce butyrate and other SCFAs. The most dramatic increases in SCFA-producing microbes were observed in lean participants, who initially had significantly lower levels of these microbes in their guts. Obese participants showed only modest increases in the proportion of SCFA-producing microbes. The ratios of different microbes in the gut also varied between lean and obese participants at every stage of the study (32). These observations highlight that host metabolic status may influence the magnitude of exercise-induced microbiota responses. Myokines released from contracting skeletal muscles, alongside SCFAs produced by the microbiota, mediate communication between these two systems (175). SCFAs provide a protective effect by reducing inflammation and promoting intestinal barrier integrity (155). Butyrate, in particular, is a primary fuel source for colonocytes and modulates the expression of more than 20% of human genes (176). This muscle–microbiota–metabolite axis offers a mechanistic explanation for systemic benefits of regular physical activity. High fitness strongly correlates with enhanced SCFA production. Athletes exhibited enriched profiles of microbial-derived SCFAs, specifically acetate, propionate, and butyrate, in their feces compared to controls (146, 156). These SCFAs contribute approximately 10% of the daily caloric requirements in humans (146, 171) and possess anti-inflammatory and anti-carcinogenic properties, directly nourishing colonocytes (171). Athletes generally showed the highest mean abundance across metabolic categories such as carbohydrate biosynthesis and energy metabolism (156). Athletes often consume different diets compared to sedentary individuals, frequently ingesting more calories, protein, fat, and carbohydrates (148). In cross-sectional studies, isolating the effects of exercise from diet is challenging (145). For example, the high microbial diversity found in rugby players correlated positively with dietary protein consumption (148, 156). Dietary extremes associated with specific sports, such as the high-protein, high-fat, low-fiber diet consumed by bodybuilders, were linked to decreased diversity and reduced abundance of beneficial SCFA-producing bacteria, like *Bifidobacterium* and *Eubacterium hallii*, compared to controls (146, 177). These data reinforce diet as a major confounder when interpreting athlete microbiome profiles. However, intervention studies maintaining a habitual diet have demonstrated that exercise alone can modulate the gut microbiota composition (e.g., increase in *Akkermansia*), establishing that physical activity independently impacts the gut microbiome (145, 170). If a moderate intensity exercise generally improves health biomarkers, reduces inflammation conversely an irregular, exhaustive, or prolonged, high-intensity training has been shown to have a deleterious influence on intestinal function (146). This again supports a dose-dependent model, in which excessive training load may shift adaptations toward maladaptive responses. This intensity can lead to increased intestinal permeability (leaky gut syndrome) and oxidative stress, potentially promoting dysbiosis and inflammation (147, 157). Changes observed immediately after ultra-endurance events include a decrease in beneficial species like *Bacteroides* and *Subdolingranulum*, with a concomitant increase in potentially pathogenic genera like *Haemophilus*, *Veillonella*, and *Streptococcus* species (147, 178, 179). These transient shifts may contribute to GI symptoms observed after extreme endurance events. It has been demonstrated that the exercise-microbiota relationship is complex, often intertwined with dietary habits (148, 149). Although numerous studies report exercise-associated changes in gut microbiota composition, findings remain heterogeneous. While several investigations demonstrate increased microbial diversity and enrichment of SCFA-producing taxa, other intervention studies have reported minimal or no detectable changes. This variability likely reflects differences in exercise intensity, duration, dietary habits, baseline fitness status, and analytical methodologies.

In conclusion, the relationship between physical activity and the gut microbiota represents a dynamic and complex area of research (Figure 1). Physical exercise, when practiced at a moderate intensity and on a regular basis, is a modifiable factor capable of promoting a favourable microbial composition, characterized by greater diversity and an increased presence of beneficial taxa. However, it is essential to consider that these effects may be temporary and require continuous stimulation, and that excessive intensity can have counterproductive effects. Diet remains an important confounding factor that interacts with the effects of exercise, suggesting the need for integrated approaches to optimize gut microbiota health. This integrated perspective is particularly relevant for GI disorders—and for celiac disease—where diet is central to management and microbiota alterations may contribute to persistent symptoms and immune activation.

**Figure 1:**
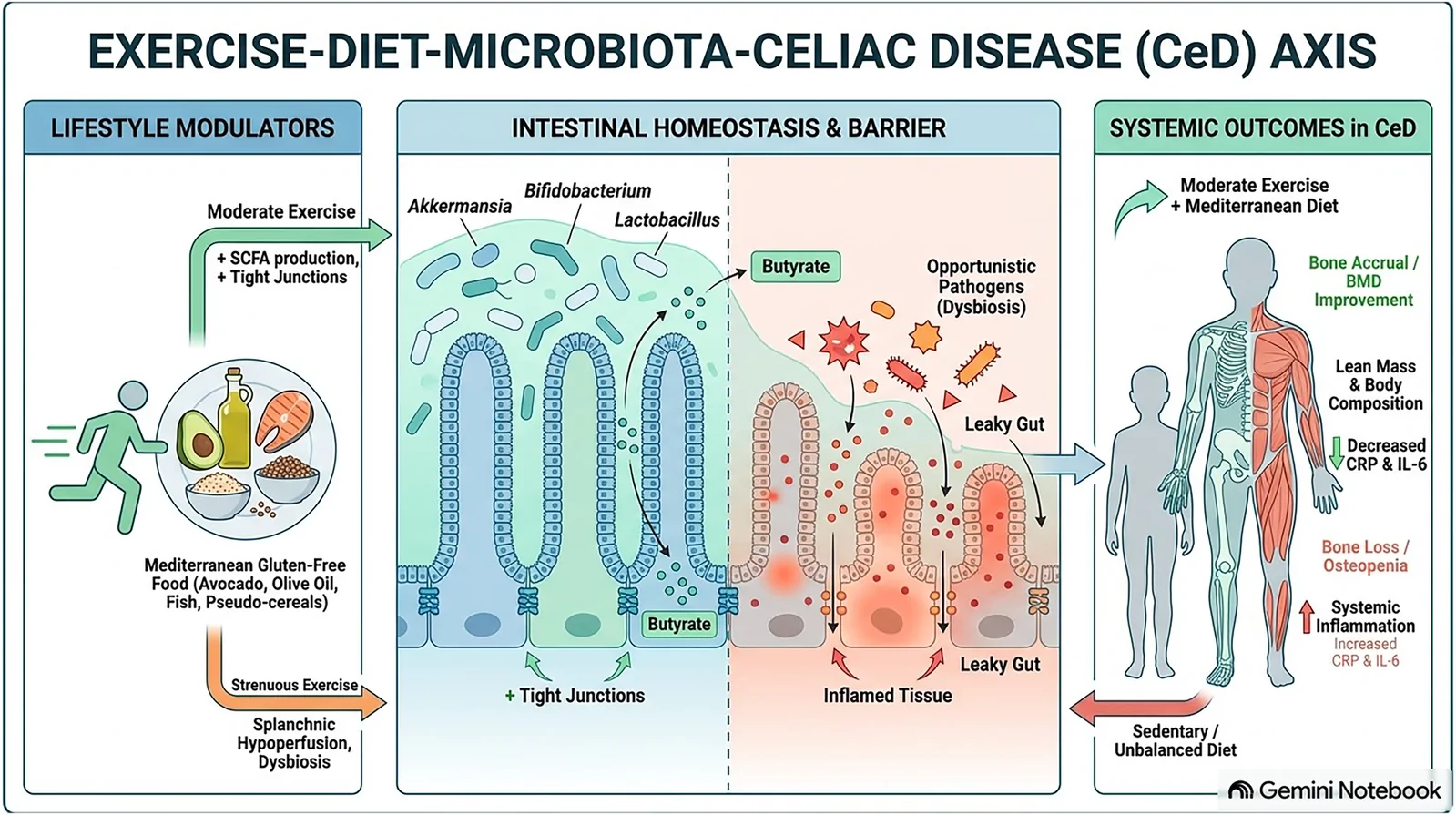
The synergistic exercise-diet-microbiota axis in celiac disease (CeD). (left) Lifestyle Modulators: Physical activity acts in a dose-dependent manner where moderate exercise is protective of the gut, while strenuous training triggers splanchnic hypoperfusion and barrier injury. Nutritional inputs range from a standard, often nutritionally unbalanced gluten-free diet (GFD) to an optimized Mediterranean-style GFD or temporary low-FODMAP restrictions. (middle) Intestinal Homeostasis: Moderate exercise and dietary fibers enrich healthy short-chain fatty acid (SCFA)-producing microbiota (e.g., *Faecalibacterium, Roseburia, Bifidobacterium*) and stimulate protective muscle-derived myokines (Irisin, IL-6) to support tight junction integrity. Conversely, active celiac enteropathy and strenuous training drive villous atrophy, epithelial damage (“leaky gut”), and mucosal inflammation. (right) Systemic Outcomes: A positive synergistic lifestyle (moderate activity + Mediterranean GFD) optimizes pediatric bone mineral density (BMD), increases lean body mass, and suppresses systemic low-grade inflammatory markers like CRP and IL-6. Conversely, sedentary behavior paired with dietary deficiencies perpetuates muscle wasting, osteopenia, chronic fatigue, and persistent gastrointestinal symptom burdens.

### 5.2 Mediterranean diet and physical activity in celiac disease

Celiac disease is characterized by low body weight, height, and low bone mineral density (BMD). These alterations reflect the systemic impact of chronic malabsorption and inflammation. Due to calcium and vitamin D malabsorption, at diagnosis, patients with celiac disease present more commonly osteoporosis and osteopenia (180, 181). A strict adherence to the gluten-free diet (GFD) normalizes body weight and BMD, beyond restoring intestinal atrophy and improving nutrient absorption. However, dietary normalization does not necessarily equate to optimal nutritional balance. Indeed, a gluten-free diet is considered nutritionally unbalanced. Through the analysis of nutrient content in cereal-based gluten-free products, researchers showed that they contain inadequate fat, protein, sugar, and fiber, and are deficient in micronutrients such as iron, zinc, magnesium, calcium, folate, vitamin D, and vitamin B12 (182). These nutritional gaps may have important implications for long-term metabolic and skeletal health. Moreover, authors administered a semi-quantitative 78-item food frequency questionnaire (FFQ) assessing all foods consumed during the usual five daily meals (breakfast, morning snack, lunch, afternoon snack, and dinner) to children with CeD who followed a GFD for at least 6 months, those who followed a GFD for less than 6 months, and healthy children. Results showed that children with CeD who followed a GFD for at least 6 months had a higher intake of vitamin B12, meat derivatives, and fish compared with those who followed a GFD for less than 6 months (all *p* < 0.05). The healthy control group showed a higher consumption of folate, iron, magnesium, selenium, and meat derivatives than children with CeD regardless of GFD duration (183). These findings indicate that dietary quality in celiac patients may remain suboptimal even after long-term adherence to a GFD.

Therefore, it is recommended that celiac patients follow a Mediterranean diet, which by definition is both a lifestyle and a dietary pattern characterized by a high consumption of plant-based foods such as fruits, vegetables, whole grains, and legumes, alongside fish, some poultry, limited red meat, and primarily unsaturated vegetable oils like extra-virgin olive oil. The macronutrient composition is variable and generally consists of 45%–55% carbohydrates, 25%–35% fat, and 15%–20% protein, with a high content of fiber and omega-3 fatty acids. This dietary pattern aligns with recommendations aimed at reducing systemic inflammation and supporting gut health.

In particular, a Mediterranean gluten-free diet is characterized by a high consumption of pseudo-cereals such as buckwheat, amaranth, quinoa, and chia, as well as cereals like teff, sorghum, rice, and maize (184). These foods may help improve micronutrient intake while preserving strict gluten avoidance.

In light of the above, a healthy diet must be accompanied by adequate physical activity, which improves body composition and contributes to the prevention of osteoporosis (138). Diet and physical activity should therefore be viewed as synergistic components of celiac disease management. The term “adequate” refers to a balanced combination of vigorous and light physical activity. Indeed, in celiac disease—as well as in GI disorders in general—regular, moderate-intensity aerobic exercise, including walking, cycling, and swimming, has demonstrated beneficial effects on GI health by improving intestinal motility and reducing constipation symptoms. These benefits complement the metabolic and musculoskeletal effects of dietary interventions. Practices such as yoga and Tai Chi further alleviate symptoms and reduce stress (85). Mind–body exercises may be particularly useful in addressing the psychosocial dimension of chronic dietary restriction.

From a practical perspective, athletes with celiac disease require careful nutritional planning to ensure adequate energy availability, optimal recovery, and sufficient intake of key micronutrients such as iron, calcium, vitamin D, and B vitamins. The combination of increased metabolic demands, potential malabsorption, and dietary restrictions may predispose physically active individuals to fatigue, impaired recovery, reduced training adaptations, and suboptimal athletic performance if nutritional intake is not appropriately monitored.

### 5.3 Low-FODMAP dietary intervation in celiac disease patient with persistent GI complaints

Recent research has increasingly focused on the role of the low-FODMAP (Fermentable Oligosaccharides, Disaccharides, Monosaccharides, and Polyols) diet (LFD) as an adjunctive strategy for patients with celiac disease (CeD) who continue to experience GI symptoms despite strict adherence to a gluten-free diet (GFD). This clinical scenario represents a frequent and challenging condition in daily practice, particularly in patients with otherwise well-controlled disease. Several studies have highlighted that these symptoms, which are not explained by ongoing gluten exposure or mucosal damage, suggest the involvement of other dietary factors—most likely fermentable carbohydrates.

A recent systematic review by Lusetti et al. synthesized all available clinical evidence and suggested that the introduction of a low-FODMAP diet can serve as a valuable short-term intervention to effectively alleviate these residual symptoms in treated celiac patients (185). In line with this, a randomized controlled trial conducted by van Megen et al. provided robust evidence that a moderately low-FODMAP diet significantly reduced GI symptoms and improved disease-specific quality of life in treated celiac patients with persistent complaints (186). These findings strengthen the clinical relevance of dietary fermentable carbohydrate restriction.

Interestingly, the observed benefits occurred without changes in celiac serology or nutritional parameters, indicating that symptom relief was not related to residual gluten exposure but rather to the restriction of fermentable carbohydrates. This distinction is crucial to avoid misattribution of symptoms to gluten exposure alone. Further supporting these findings, a couple of studies reviewed the broader application of the low-FODMAP diet across gluten-related disorders, including non-celiac gluten sensitivity (NCGS) (187–190). They proposed that many individuals who report symptom improvement after gluten withdrawal might actually respond to the reduction of FODMAPs, particularly wheat fructans, rather than to the elimination of gluten itself. This observation challenges traditional interpretations of “gluten sensitivity”. This observation reinforces the idea that, in some cases, symptoms attributed to “gluten sensitivity” could be driven by carbohydrate malabsorption. The authors concluded that, while the gluten-free diet remains essential for managing celiac disease, the low-FODMAP approach can be considered a useful complementary tool to control functional GI symptoms. Nevertheless, they noted that current studies are small and heterogeneous, calling for larger randomized trials to confirm these preliminary findings.

However, potential downsides of prolonged FODMAP restriction have also been reported. In a randomized trial, Herfindal et al. explored the effects of the low-FODMAP diet on the gut microbiota of treated celiac patients and found that although the diet significantly improved bloating and abdominal discomfort, it also reduced beneficial bacterial taxa such as *Bifidobacterium* and altered short-chain fatty acid production (188, 189). These microbiota changes mirror those observed in other restrictive dietary patterns. These findings underscore the need for dietary reintroduction phases and professional dietetic guidance to preserve microbial diversity and nutritional adequacy.

For athletes and physically active individuals with celiac disease, optimal management requires balancing symptom control, nutritional adequacy, gut microbiota preservation, and the metabolic demands imposed by exercise. Consequently, dietary interventions should not focus exclusively on symptom reduction but also on maintaining adequate nutritional support for training, recovery, and long-term health. In fact, persistent gastrointestinal symptoms such as bloating, abdominal pain, urgency, and diarrhea can negatively affect exercise tolerance, training continuity, competition performance, and overall quality of life. Reducing symptom burden through a short-term low-FODMAP approach may therefore facilitate greater participation in physical activity and improve exercise-related comfort in selected athletes. However, caution is warranted in athletic populations. Restrictive dietary patterns may inadvertently reduce carbohydrate availability, fiber intake, and dietary diversity, potentially affecting energy balance, recovery processes, and exercise performance. Therefore, low-FODMAP interventions should be individualized, periodically reassessed, and implemented under the supervision of experienced healthcare professionals, particularly in athletes with high training volumes and elevated nutritional requirements.

Therefore, current evidence suggests that while the gluten-free diet remains the only causal treatment for celiac disease, the low-FODMAP approach represents a promising adjunctive strategy to manage residual functional symptoms, provided it is implemented carefully and with appropriate medical supervision.

## 6 Clinical implications and take-home messages

Current evidence does not support a validated CeD-specific frequency, intensity, time, and type (FITT) prescription stratified by age or athletic level. A recent systematic review identified a limited and heterogeneous evidence base, with substantial variation in study design, population, and physical activity assessment or intervention (191). The framework below should therefore be interpreted as a pragmatic adaptation of international age-specific physical activity recommendations rather than as a disease-specific exercise prescription (162). The quantitative targets are derived from World Health Organization recommendations, whereas the CeD-specific considerations represent a cautious clinical overlay informed by the nutritional, gastrointestinal, and skeletal concerns discussed throughout this review.

For adolescent competitive athletes, the values shown above represent general health targets rather than a sport-specific performance prescription or an upper limit for training volume (162). No controlled study has established an optimal frequency, intensity, duration, or type of exercise for adolescent athletes with CeD (191). Training beyond these general health targets should therefore be individualized according to the demands of the sport and the athlete's clinical and nutritional status. This final recommendation represents a pragmatic clinical approach and should not be interpreted as a CeD-specific protocol supported by intervention trials.

Recommendations should also be considered according to activity level. Recreationally active individuals with treated and clinically stable CeD may generally follow age-appropriate physical activity recommendations (162). Elite and competitive athletes may require closer management of training load, recovery, gastrointestinal symptoms, and nutritional adequacy because prolonged, high-intensity exercise can increase gastrointestinal stress and make adequate gluten-free fuelling more challenging (2, 13, 127, 128). However, no study has directly compared exercise-related outcomes between elite and recreationally active individuals with CeD; therefore, this distinction represents a pragmatic, risk-based approach rather than evidence of different exercise effects.

- Physical activity should be considered a core component of celiac disease management, alongside strict gluten avoidance.
- Exercise prescriptions must be individualized, by taking into account disease activity, symptom profile, age, and training goals.
- Moderate-intensity aerobic and resistance training are generally beneficial, while excessive or prolonged high-intensity exercise may exacerbate GI symptoms.
- A Mediterranean-style gluten-free diet can help address nutritional imbalances and support bone and metabolic health.
- In patients with persistent functional GI symptoms, a low-FODMAP diet may be used as a short-term adjunct under professional supervision.
- Preservation of gut microbiota diversity should be a key consideration when implementing restrictive dietary interventions.
- Pediatric patients require special focus to optimize growth, bone health, and long-term outcomes.
- Gluten-free diet alone may not fully address all clinical and functional aspects of celiac disease.
- Physical activity acts as a powerful, bidirectional modulator of GI health, immunity, and the gut microbiota.
- The benefits of exercise follow a dose-dependent pattern: moderation and individualization are essential.
- Diet and exercise should be viewed as synergistic, not independent, components of disease management.
- Precision lifestyle interventions offer a promising avenue to improve quality of life and performance in celiac patients.

## 7 Conclusions

Physical activity exerts profound and multifaceted effects on GI physiology, immune regulation, metabolic balance, and psychological wellbeing. Across both the general population and athletic settings, regular moderate-intensity exercise is consistently associated with improved GI motility, reduced systemic inflammation, enhanced gut microbiota diversity, and overall health benefits. Conversely, excessive or poorly individualized high-intensity training may adversely affect GI function through mechanisms such as splanchnic hypoperfusion, increased intestinal permeability, oxidative stress, and transient dysbiosis. These observations underscore the need for carefully tailored exercise prescriptions that balance performance goals with GI health.

Dietary patterns and gut microbiota composition emerge as key modulators of exercise-related GI responses. Nutritional quality, macronutrient composition, and fermentable carbohydrate intake interact closely with physical activity to shape intestinal homeostasis, immune signaling, and symptom expression. Importantly, these interactions are relevant not only to athletes but to all physically active individuals, highlighting lifestyle interventions as central components of GI health maintenance.

Within this broad framework, CeD represents a distinctive clinical model in which the interplay between physical activity, diet, and gut microbiota becomes particularly evident. As a condition characterized by immune-mediated enteropathy and lifelong dietary restriction, CeD amplifies the physiological and nutritional challenges associated with physical activity. While strict adherence to a gluten-free diet remains the only causal therapy, dietary treatment alone may not fully address persistent GI symptoms, impaired bone health, altered body composition, or reduced quality of life, especially in physically active individuals and athletes.

In this context, appropriately prescribed physical activity may serve as a valuable non-pharmacological adjunct in CeD management, contributing to improvements in musculoskeletal health, inflammatory status, psychological wellbeing, and functional performance. At the same time, individuals with CeD - particularly athletes - face unique challenges related to increased energy demands, micronutrient deficiencies, and symptom persistence, necessitating individualized exercise and nutritional strategies.

Emerging evidence further supports the role of exercise-induced modulation of the gut microbiota as a potential mechanism linking physical activity to gastrointestinal and systemic health outcomes. However, these adaptations appear to be highly dependent on exercise intensity, training chronicity, dietary composition, and host characteristics, reinforcing the importance of integrated and personalized lifestyle approaches.

Pediatric populations warrant special consideration, as childhood and adolescence represent critical periods for growth, bone accrual, and long-term health. In children and adolescents with CeD, the combination of optimized dietary patterns and appropriate physical activity is essential to support peak bone mass achievement and prevent future skeletal complications.

In conclusion, physical activity should be regarded as a central, modifiable determinant of GI health across populations. CeD offers a clinically relevant model in which the interactions between exercise, diet, and gut microbiota are particularly pronounced, emphasizing the importance of individualized, multidisciplinary strategies. Integrating personalized physical activity programs with tailored dietary interventions represents a promising approach to improving GI function, overall wellbeing, and quality of life in both the general population and individuals living with CeD.
